# What Is Next for Connexin and Pannexin?

**DOI:** 10.1159/000533281

**Published:** 2023-08-16

**Authors:** Timothy M. Sveeggen, Anna Kosmach, Pooneh Bagher

**Affiliations:** Cellular and Integrative Physiology, University of Nebraska Medical Center, Omaha, NE, USA

Signaling across membranes is vital for tissue and organ function throughout the body. This is achieved through careful regulation of transmembrane channels allowing for passage of ions and second messengers to and from the cytosol and intracellular compartments, which can generate electrical impulses and trigger signaling cascades. Connexins and pannexins are among the most versatile transmembrane channel proteins, which shape cell behavior and tissue function with extraordinary finesse. In this volume, pannexins and connexins 37, 40, and 43 are reviewed [1–4], with special consideration for tissue specificity of expression, cell type-specific functions, and their roles in development and disease.

## Background of Connexins and Pannexins

**Fig. 1.**
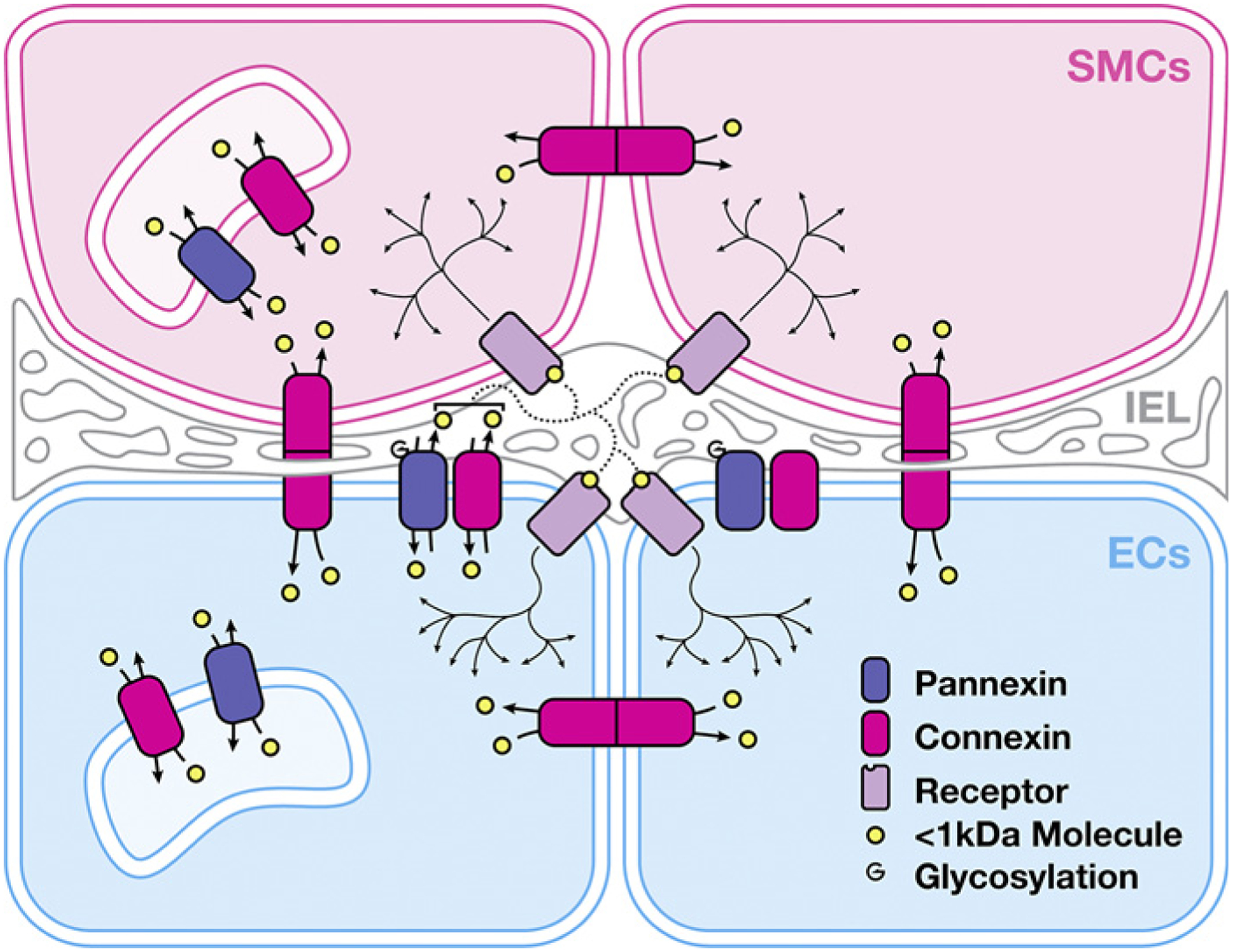
Schematic of connexin and pannexin channel-mediated communication pathways in smooth muscle and endothelial cells. Connexin hemichannels and gap junctions (magenta) as well as pannexin channels (dark blue) are localized to membranes of organelles and the plasma membrane. Molecules <1 kDa (yellow) traverse channels and can bind possible target receptors (lavender, dotted line) via paracrine and/or autocrine mechanisms, activating downstream signaling pathways (solid arrows). Molecules <1 kDa traverse gap junctions via homocellular connections shown here between endothelial cells (ECs, blue) and between smooth muscle cells (SMCs, pink). Molecules <1 kDa traverse gap junctions via heterocellular connections at myoendothelial junctions (MEJs). MEJs are microdomains where EC projections through holes in the internal elastic lamina (IEL, gray) interact directly with adjacent vascular SMCs via gap junctions. Molecules <1 kDa can also traverse channels in intracellular compartments. The extracellular N-glycosylation of pannexins which prevents formation of pannexin gap junctions is denoted by “G.”

The term “connexin” first appeared in the literature in 1974 [5] and was named as such due to the ability of connexins to “connect” adjacent cells via a “nexus” (of the Latin origin “to bind”) [6]. The first connexin was not cloned until 1986 [7], but since then the field has rapidly expanded due to identification of 21 different human connexins with tissue-specific roles [8]. Of note, four connexin proteins (Cx): Cx37, Cx40, Cx43, and Cx45 are expressed and play a crucial functional role in blood vessels [2–4]. Gap junctions form when six-connexin oligomers, collectively known as a hemichannel or connexon, bind with another hemichannel on an adjacent cell to make a single nonselective pore that crosses both plasma membranes [9]. It should be noted that connexin hemichannels function both individually and when coupled to an adjacent hemichannel to form a gap junction. Thus, gap junctions allow for physical and electrical coupling between adjacent cells of both homologous (homocellular) or differing (heterocellular) cell types, while hemichannels allow for signaling across intracellular and extracellular spaces (Fig. 1).

Pannexins were discovered in 2000 [10], and since their discovery, three pannexin proteins: PANX1, PANX2, and PANX3 have been identified and are known to be expressed in the blood vasculature [1]. Pannexins were originally believed to form gap junctions like connexins, due to their predicted protein sequence homology with invertebrate gap junction proteins [10]. One key feature distinguishing pannexins from connexins, however, is that pannexin channels do not typically form gap junctions [11]. Pannexins similarly oligomerize (6 or 8 monomers) to form transmembrane channels [12]; however, extracellular N-glycosylation of pannexin prevents linkage between pannexin channels of neighboring cells (Fig. 1) [13, 14]. Indeed, prevention of this N-glycosylation in vitro does allow pannexin gap junctions to form [14, 15]. Although pannexins form nonselective pores, they are widely recognized for their role in releasing ATP into the extracellular compartment, which can then stimulate adjacent purinergic receptors in both a paracrine and autocrine fashion (Fig. 1) [1, 16].

Both connexins and pannexins form nonselective channels with large pores, meaning that any molecule ~1 kDa or smaller could theoretically traverse the pore lumen (Fig. 1). Since >35,000 metabolites fall within this exclusion limit [9], connexins and pannexins may signal via a wide range of messengers and, thus, can be involved in a large variety of cellular functions. As connexin and pannexin channels can localize to intracellular membranes, the plasma membrane, and to specific microdomains, they play a key role in the influx and efflux of signaling molecules (Fig. 1). While one might suspect this means connexins and pannexins serve redundant functions, in reality each type of connexin and pannexin has varying pore permeabilities, posttranslational modifications, channel gating dynamics, intracellular localization, and cell-type expression profiles. Thus, despite their general similarities, connexins and pannexins provide specialized signaling to the tissues in which they are expressed.

## Connexins and Pannexins in the Vasculature and Beyond

Cx37 is primarily expressed in the endothelium, comprising gap junctions between endothelial cells (ECs) and within gap junctions at the myoendothelial junction, a specialized microdomain where ECs send out projections and interact directly with adjacent vascular smooth muscle cells (SMCs) [2]. Cx37 is highly sensitive to flow-dependent signals, and the review by Fang and Burt discusses the role of Cx37 and its various phosphorylation states in cell cycle control, nitric oxide (NO)-dependent modulation of vascular tone, and further details how Cx37 can be upregulated in SMCs to reduce medial thickening following injury [2].

Cx40 expression varies throughout the vasculature and has been found in both ECs and SMCs, but it is thought to be predominately expressed in ECs, and much like Cx37, Cx40 is expressed between ECs and in myoendothelial junctions [4]. In the review by Márquez et al. [4], the role of Cx40, in combination with other connexin proteins, in the development of the cardiovascular system is discussed. Further, they explore the essential role for Cx40 in conducted vasodilation, a mechanism whereby electrotonic signals propagate between cells of the vascular wall, primarily through gap junctions, to ensure coordinated changes in diameter within a vascular network [17]. They highlight that Cx40 plays a dual role in blood pressure regulation through its direct actions in the endothelium and by inhibiting renin release from juxtaglomerular cells.

Cx43 differs slightly from Cx37 and Cx40 in that it is expressed in pericytes, SMCs, ECs, and the junctions between these cell types, with the cellular expression pattern differing along the arterial tree – from conduit arteries down to capillaries [3]. Sedovy et al. [3] explores the nuances of Cx43 function across vessel types and vascular beds, further describing how Cx43 can modulate restenosis/neointimal formation, vasoreactivity, permeability, and angiogenesis. They also highlight the role of Cx43 in disease states such as atherosclerosis, hypertension, and diabetes.

Pannexins are widely expressed in numerous cell types but are known to play an important role in the vasculature with PANX3, PANX2, and PANX1 expression observed in ECs, SMCs, and both ECs/SMCs, respectively [1]. The review by Wakefield and Penuela discusses the role of pannexins in the vasculature, skeletal muscle, cartilage, bone, and adipose tissue with a particular emphasis on the relationship between exercise and pannexin expression/function [1].

The reviews in this current volume demonstrate that connexins and pannexins play fundamental roles as transmembrane channels that can impact the physiology both within a cell and collectively within tissue and organ systems. By further understanding the unique attributes of connexins and pannexins under physiological and pathophysiological conditions, their clinical importance and therapeutic potential will continue to emerge.

## What Is Next?

While much has been learned over the last several decades in regard to the various roles of connexins and pannexins in the vasculature and beyond, there remains much more to discover. Disruption of the expression and/or function of a particular connexin or pannexin is known to activate compensatory mechanisms (e.g., upregulation of other connexins/pannexins, alterations in subcellular localization) [18]. Thus, there are pathways including, but not limited to, the regulation of channel gating, transcription, and subcellular localization of connexins and pannexins that need to be further explored, in particular during pathology and physical stress, such as exercise. The reviews in this current volume [1–4] expand on these research areas and highlight the gaps in knowledge that need to be investigated before the targeting of connexins and pannexins can be fully translated from bench to bedside. There also remains technological challenges that need to be overcome to fully harness the therapeutic potential of connexins and pannexins. These include the following: (1) How to target a specific connexin or pannexin in vivo and (2) how to target connexins and/or pannexins in a cell- and/or tissue-specific manner? It is only with further research into the nuanced signaling of connexins and pannexins and technological advancements in targeted therapeutic approaches (e.g., drug delivery, gene therapy) that applications can be developed for the prevention and treatment of connexin- and pannexin-related pathologies.
